# PP98 Cost Effectiveness Of Positron Emission Tomography-Computed Tomography In The Detection Of Metastases Originating From Oral Squamous Cell Carcinoma

**DOI:** 10.1017/S0266462325103164

**Published:** 2025-12-29

**Authors:** Daniele Marques, Luciene Bonan, Kátia Marie Simões e Senna, Katia Senna

## Abstract

**Introduction:**

Oral squamous cell carcinoma (OSCC) is a public health issue due to late diagnosis, often caused by its initial silent manifestation and economic barriers. This leads to higher rates of metastasis and recurrence. Diagnosis is made through biopsy, followed by staging using imaging techniques such as neck or chest computed tomography (CT), neck magnetic resonance imaging (MRI), and positron emission tomography-computed tomography (PET-CT) for metastasis detection.

**Methods:**

A cost-effectiveness analysis was conducted using a decision tree model with a one-year time horizon and no discount rate applied. The model included estimates of direct costs related to OSCC management and used correct diagnosis as the main outcome. The approach was based on a comparative evaluation of costs and clinical outcomes, considering the resources available in the healthcare system. Data were extracted from reliable sources, ensuring the robustness of the results and their applicability to public health practices related to the management of patients with this neoplasm.

**Results:**

PET-CT had an incremental cost of USD362.54, an incremental effectiveness of 0.17, and an incremental cost-effectiveness ratio (ICER) of USD2,122.33 for each correct diagnosis. PET-CT was classified as a non-dominated strategy, meaning it is economically viable for improving case identification. In contrast, neck MRI combined with chest CT had an ICER of −USD1,969.85 per correct diagnosis, indicating that it is a dominated strategy—less efficient and with an unfavorable cost-benefit when compared with other diagnostic alternatives.

**Conclusions:**

PET-CT for detecting cervical and distant metastases in patients with OSCC had higher effectiveness but incurred greater costs than clinical strategies such as neck and chest CT or neck MRI combined with chest CT. While more precise, PET-CT is less favorable economically, presenting a trade-off between accuracy and cost.

